# Inactivation of interleukin-15 reduces spontaneous atherosclerosis in *apolipoprotein E*-deficient mice

**DOI:** 10.3389/fphys.2026.1859434

**Published:** 2026-08-04

**Authors:** Omid Dadoo, B. Sumayyah H. Sokeechand, Anna Lee, Mark T. Fuller, Yu Chang David Wang, Melissa E. MacDonald, Nicole L. Batenburg, Suleiman A. Igdoura, Carl D. Richards, Ali A. Ashkar, Bernardo L. Trigatti

**Affiliations:** 1 Department of Biochemistry and Biomedical Sciences, McMaster University, Hamilton, ON, Canada; 2 Thrombosis and Atherosclerosis Research Institute, Hamilton Health Sciences and McMaster University, Hamilton, ON, Canada; 3 Centre for Metabolism, Obesity and Diabetes Research (MODR), McMaster University, Hamilton, ON, Canada; 4 Department of Biology, McMaster University, Hamilton, ON, Canada; 5 Department of Pathology and Molecular Medicine, McMaster University, Hamilton, ON, Canada; 6 Department of Medicine, McMaster University, Hamilton, ON, Canada; 7 McMaster Immunology Research Centre, McMaster University, Hamilton, ON, Canada

**Keywords:** antibody-based therapeutic, atherosclerosis, inflammation, interleukin-15 (IL-15), knockout mouse

## Abstract

**Introduction:**

Interleukin (IL)-15 is essential for the survival and maturation of natural killer (NK) and CD8^+^ T cells, and it directly activates macrophages.

**Methods:**

In the present study, we examined the effects of inactivating *Il-15* on atherosclerosis in *apolipoprotein (apo) E*-deficient mice.

**Results and discussion:**

As expected, *Il-15* deficiency reduced circulating NK and CD8^+^ T cells in *ApoE^−/−^* mice. It also increased body weights in female but not male *ApoE^−/−^* mice and increased plasma total cholesterol levels in both. Despite this, the *Il-15* knockout reduced spontaneous atherosclerotic plaque development in both male and female *ApoE^−/−^* mice (fed a normal diet) at 25 weeks of age, and in female normal diet-fed *ApoE^−/−^* mice at 15 weeks but not at 38 weeks of age. Furthermore, *Il-15* knockout did not impact the levels of atherosclerosis in 25-week-old female *ApoE^−/−^* mice fed a high-fat, high-cholesterol diet for 15 weeks. However, the 6-week treatment with an antibody (M96) that blocks IL-15’s interaction with the IL-2Rβγ_c_ complex but does not interfere with its interaction with IL-15Rα reduced spontaneous atherosclerosis in female *ApoE^−/−^* mice. *ApoE* knockout mice in which IL-15 was inactivated or neutralized with an antibody exhibited reduced accumulation of CD11b^+^ and CD8^+^ cells within atherosclerotic plaques. These findings demonstrate that interfering with IL-15 signaling through the IL-2Rβγ_c_ complex delays spontaneous atherosclerosis development in ApoE-deficient mice.

## 1 Introduction

Atherosclerosis is a chronic inflammatory disease involving monocyte infiltration of the arterial intima, differentiation into phagocytic macrophages, engulfment of modified lipoproteins such as oxidized low-density lipoproteins, and formation of lipid-engorged foam cells—hallmark cellular components of atherosclerotic plaques (Smith et al., 1995; Hansson and Libby, 2006; Björkegren and Lusis, 2022). Other leukocytes, including natural killer (NK) cells, NK T cells, and CD8^+^ T cells, promote atherosclerosis by secreting pro-inflammatory cytokines and cytotoxic molecules such as perforin and granzyme-B, which induce apoptosis of cells within the atherosclerotic plaques (Tupin et al., 2004; Whitman et al., 2004; Rogers et al., 2008; Kyaw et al., 2013; Selathurai et al., 2014; Björkegren and Lusis, 2022). Inflammatory regulators such as C-X-C motif chemokine ligand 9 [CXCL9; also known as monokine induced by gamma interferon (MIG)], monocyte chemoattractant protein-1 (MCP-1), and regulated upon activation, normal T cell expressed and secreted (RANTES) act as chemoattractants, facilitating immune cell recruitment to sites of inflammation (Pattison et al., 1996; Gu et al., 1998; Szentes et al., 2018). Cytokines such as interferon-gamma (IFN-γ), interleukin-1β, and tumor necrosis factor-α activate macrophages, promote foam cell formation, and enhance T-cell proliferation within atherosclerotic plaques. These cytokines also induce macrophage apoptosis and amplify cytokine production, contributing to plaque progression and destabilization (Ait-Oufella et al., 2011; Boshuizen and De Winther, 2015; González et al., 2022).

Interleukin-15 (IL-15) is a 14-kDa cytokine with several immunomodulatory functions (Budagian et al., 2006). Macrophages and dendritic cells secrete IL-15, which is presented by its cognate receptor, IL-15 receptor α (IL-15Rα), to a heterodimeric receptor complex consisting of IL-2 receptor β (IL-2Rβ) and IL-2Rγ (also known as the common gamma chain receptor, γ_c_) on NK cells, NK T cells, and CD8^+^ T cells (Giri et al., 1994; Giri et al., 1995; Fehniger and Caligiuri, 2001; Budagian et al., 2006). This promotes the differentiation, proliferation, survival, activation, and homing of these cytotoxic CD8^+^ T cells, NK cells, and NK T cells (Carson et al., 1994; Kennedy et al., 2000; Fehniger and Caligiuri, 2001; Berard et al., 2003; Burkett et al., 2004; Mortier et al., 2009; Huntington et al., 2011). Genetic inactivation of IL-15 in mice results in substantial decreases in the abundance of these cells and in their recruitment into tissues (Kennedy et al., 2000; Ashkar et al., 2003; Ashkar et al., 2009; Barra et al., 2010; Barra et al., 2014; White et al., 2017). IL-15 also upregulates perforin, granzyme B, and IFN-γ expression in cytotoxic CD8^+^ T cells, enhancing their cytotoxic function (Liu et al., 2002; Cheuk et al., 2017; Nolz and Richer, 2020). Additionally, IL-15 signals through IL-15Rα in macrophages, inducing the expression of pro-inflammatory and pro-atherogenic genes, including RANTES (Chenoweth et al., 2012).

IL-15 has been detected in both human and mouse atherosclerotic plaques, with elevated serum IL-15 in patients with cardiovascular diseases compared to healthy individuals (Wuttge et al., 2001; Gokkusu et al., 2010; van der Meer et al., 2011; Panigrahi et al., 2020). In human atherosclerotic plaques, IL-15 was associated with increased CD8^+^ cytotoxic T-cell recruitment into plaques (Panigrahi et al., 2020). Antibody-mediated blockade of IL-15 was reported to enhance neo-intima formation in mice following carotid arterial injury using a perivascular cuff, suggesting that IL-15 may suppress vascular injury associated with atherosclerosis (Cercek et al., 2006). Conversely, vaccination of LDL receptor-deficient (*Ldlr^−/−^*) mice against IL-15 reduced carotid artery atherosclerosis in mice fed a high-fat diet following perivascular cuff placement, despite an increase in the numbers of macrophages detected in the artery wall (van Es et al., 2011). The same study also reported an increase in circulating CD8^+^ T cells; however, the effect of IL-15 neutralization on CD8^+^ cell recruitment into atherosclerotic plaques was not examined (van Es et al., 2011). Therefore, the role of IL-15 in regulating atherosclerosis progression and macrophage and CD8^+^ T cell infiltration into plaques remains to be fully elucidated.

In this study, we directly tested the role of IL-15 on atherosclerosis by examining the impact of *Il-15* gene deletion (Kennedy et al., 2000) in *apolipoprotein E*-deficient (*ApoE^−/−^*) mice (Piedrahita et al., 1992). *Il-15* deficiency significantly reduced atherosclerosis in 15-week-old and 25-week-old *ApoE^−/−^* mice fed a normal low-fat, low-cholesterol diet, despite markedly elevated plasma cholesterol levels. The reduced atherosclerosis in female *Il-15^−/−^ApoE^−/−^* mice was accompanied by reduced monocyte chemotactic protein-1 levels and CD11b, a marker of monocyte-derived macrophages. However, the protection conferred by *Il-15* deletion was lost by 38 weeks of age or in mice fed a high-fat, high-cholesterol, cholate-containing, atherosclerosis-promoting diet. *Il-15* deletion also reduced circulating CD8^+^ T cells and NK cell levels in 25-week old male and female *ApoE^−/−^* mice and trends toward decreased CXCL9 levels. We also tested the impact of treating *ApoE^−/−^* mice with an anti-IL-15 monoclonal antibody, M96, which selectively blocks IL-15 binding to IL-2Rβγ_c_ complex but not to IL-15Rα (Lebrec et al., 2013). We found that M96 treatment significantly reduced atherosclerosis, circulating CD8^+^ T cells, NK cells, and CD8^+^ cell abundance in the atherosclerotic wall of the aortic sinus. These findings suggest that IL-15 predominantly impacts early-stage spontaneous atherosclerosis, independently of lipid levels, and likely by directly affecting circulating CD8^+^ T cells and/or NK cells and promoting MCP-1-mediated monocyte recruitment and CXCL9-mediated CD8^+^ T-cell recruitment to atherosclerotic plaques through the IL-2Rβγ_c_ complex.

## 2 Materials and methods

### 2.1 Ethics statement

All procedures involving mice were approved by the McMaster University Animal Research Ethics Board and were in accordance with the guidelines of the Canadian Council on Animal Care.

### 2.2 Mice and diets

All mice were on a C57BL6/J background. *Il-15^−/−^* mice (Kennedy et al., 2000; Ashkar et al., 2003; Ashkar et al., 2009; Barra et al., 2010; Barra et al., 2014; White et al., 2017) were from Taconic Biosciences (Germantown, NY, USA). *ApoE^−/−^* mice were from Jackson Laboratories (Bar Harbor, ME, USA). *Il-15^−/−^ApoE^−/−^* and *Il-15^+/+^ApoE^−/−^* lines of mice were generated by mating *Il-15^−/−^* mice with *ApoE^−/−^* mice. The resulting double heterozygous mice were mated back to *ApoE^−/−^* mice to generate *Il-15^+/−^ ApoE^−/−^* mice, which were then intercrossed to generate founders of the *Il-15^−/−^ApoE^−/−^* and *Il-15^+/+^ApoE^−/−^* lines. Genotypes for the IL-15 gene were determined via separate PCR analyses using primers GAG GGC TAA ATC TGA TGC GTG TG and GAG CTG GCT ATG GCG ATG GGC for the wild-type (WT or +) allele (expected product size 210 base pairs) and GAA TGG GCT GAC CGC TTC CTC G and TCA TAT CCT CTG CAC CTT GAC TG for the knockout mutant (Mut or −) allele (expected product size 520 base pairs). Genotypes for the ApoE gene were determined via multiplex PCR analysis as described by the supplier using primers GCC TAG CCG AGG GAG AGC CG (oIMR0180: common primer for both the WT and knockout Mut alleles), TGT GAC TTG GGA GCT CTG CAG C (oIMR0181: reverse primer for the WT allele), and GCC GCC CCG ACT GCA TCT (oIMR0182: reverse primer for the knockout Mut allele). Expected product sizes were 155 and 245 base pairs for the WT and knockout Mut alleles, respectively. Mice were housed in the Central Animal Facility at McMaster University and in the David Braley Research Institute Animal Facility at Hamilton Health Sciences, with free access to food and water. All mice were fed a normal diet (Teklad TD2918: 5% fat and 0% cholesterol; Inotiv, Madison, WI, USA) unless otherwise noted. Female and male mice were analyzed separately. *Il-15^−/−^ApoE^−/−^* and *Il-15^+/+^ApoE^−/−^* mice fed the normal diet were harvested for analysis at 15, 25, or 38 weeks of age as indicated. Female mice were also fed a high-fat, high-cholesterol, cholate-containing diet (Teklad Research diet, TD88051: 15% fat, 1.25% cholesterol, and 0.5% sodium cholate; Inotiv, Madison, WI, USA) for 15 weeks beginning at 10 weeks of age.

### 2.3 Antibody-mediated neutralization of IL-15

Female *ApoE^−/−^* mice fed the normal diet were treated weekly with 25 µg of either a mouse anti-IL-15 monoclonal antibody (mAb) (M96), which neutralizes its interaction with the IL-2Rβγ_c_ receptor complex (Lebrec et al., 2013), or a control mAb (AMG245) starting at 9 weeks of age and continuing for 6 weeks, until they were euthanized and analyzed at 15 weeks of age. The antibodies, generously provided by Amgen Inc. (Thousand Oaks, CA, USA; contract number 2014589254), were injected i.p. in 100 μL of sterile saline (0.9% NaCl). Mice were fed the normal diet for the duration of the study.

### 2.4 Harvest

Mice were fasted for 12 h and were anesthetized using general isoflurane/O_2_. Heparinized blood was collected via cardiac puncture. Mice were humanely euthanized while still under anesthesia via thoracotomy, and tissues were perfused *in situ* with saline followed by formalin prior to the removal of the heart and aorta. Hearts were embedded in Cryomatrix (Thermo Scientific, Ottawa, ON, Canada).

### 2.5 Flow cytometry

Blood collected at harvest was subjected to red blood cell lysis, followed by staining with phycoerythrin (PE)-labeled NK1.1, fluorescein isothiocyanate (FITC)-labeled CD3, peridinin chlorophyll-cyanine (PerCP-Cy) 5.5-labeled CD4, allophycocyanin (APC)-H7-labeled CD8, FITC-labeled CD11b, and APC-labeled Ly6C antibodies (BD Pharmingen, Mississauga, ON, Canada). Flow cytometry was performed after the addition of 123count eBeads (Thermo Fisher Scientific Canada) using a BD LSR II flow cytometer, and data analysis was conducted using the FlowJo software. The gating strategy is shown in **Supplementary Figure 1**. Briefly, for NK, NK T, and CD8 T cells, samples were stained with NK1.1, CD3, and CD8 antibodies. Forward and side scatter plots were used to gate cells and differentiate them from 123count eBeads. Cells (gated based on forward and side scatter) were analyzed for PE-NK1.1 versus FITC-CD3 fluorescence. CD3^−^NK1.1^+^ cells were counted as NK cells, CD3^+^NK1.1^+^ cells were counted as NK T cells, and CD3^+^NK1.1^−^ cells were gated and analyzed for APC-H7-CD8a versus PerCP-Cv5.5-CD4 fluorescence to evaluate CD4^−^CD8a^+^ cells (CD8^+^ T cells). Alternatively, leukocytes stained for CD11b and Ly6C were analyzed using forward and side scatter to gate on cells. Analysis of PE-CD11b fluorescence versus APC-Ly6C fluorescence was performed to evaluate Ly6C^+^CD11b^+^ cell abundance.

### 2.6 Plasma analysis

Heparinized blood was centrifuged at 3,300 × *g* for 10 min, and plasma was collected. Plasma lipoprotein fractions were separated via fast protein liquid chromatography as described previously (Rigotti et al., 1997). Total cholesterol (Cholesterol Infinity, Thermo Scientific, Ottawa, ON, Canada) and triglyceride (Wako Diagnostics, Richmond, VA, USA) content were measured using enzymatic assay kits. Plasma cytokine levels were measured using the Mouse Cytokine Array/Chemokine Array 32-Plex (Eve Technologies, Calgary, AB, Canada).

### 2.7 Atherosclerosis analysis

Frozen sections (10-µm-thick) were collected at the aortic sinus and stained with Oil Red O and hematoxylin, followed by analysis as previously described (Trigatti et al., 1999; Kluck et al., 2023; Qian et al., 2025). Five sections per mouse were analyzed, spaced at 100-µm intervals across 400 µm of the aortic sinus, centered at the aortic root. Plaque area was plotted against the distance from the aortic sinus, and plaque volumes were calculated by determining the area under the curve of plaque cross-sectional area versus distance for each mouse using the GraphPad Prism software version 9.2.0.

### 2.8 Immunofluorescence and immunohistochemistry

Immunostaining was performed using rabbit anti-MCP-1 (Abcam, Cambridge, MA, USA), rabbit anti-smooth muscle alpha actin (Abcam, Cambridge, MA, USA), and biotinylated anti-CD11b (BD Pharmingen, Mississauga, ON, Canada) antibodies. Antigens were visualized using goat anti-rabbit conjugated to AlexaFluor 594, or streptavidin conjugated to AlexaFluor 568 (Molecular Probes, Burlington, ON, Canada). Sections were counterstained for nuclei with DAPI (Invitrogen/Molecular Probes, Burlington, ON, Canada). Immunofluorescence images were captured at ×20 magnification using a Zeiss Axiovert 200M microscope and the AxioVision software. Immunofluorescence was quantified as the area of the plaque positive for the signal of interest, relative to the entire plaque area using the ImageJ software.

Immunohistochemistry for CD8 was performed at the Histology Lab of the McMaster Immunology Research Centre. Briefly, sections were fixed in cold acetone for 15 min, blocked with 1% swine serum for 15 min, and incubated with a rat anti-CD8a antibody (BD Pharmingen, Mississauga, ON, Canada; 1:50 dilution) for 1 h. This was followed by a 1-h incubation with biotinylated rabbit anti-rat antibody (Agilent, formerly Dako, Mississauga, ON, CA) and subsequent incubation with streptavidin/horseradish peroxidase (HRP) (Agilent, formerly Dako, Mississauga, ON, Canada; 1:600 dilution) for 1 h at room temperature. Sections were then treated with an aminoethylcarbazole chromogen (Acros Organics-Fisher, Hampton, NH, USA) for 30 min. Atherosclerotic plaques were imaged at ×40 magnification using the Zeiss Axiovert 200M inverted microscope (Carl Zeiss Canada, Ltd., Toronto, ON, Canada), which was also used to quantify the number of CD8^+^ cells in the plaques.

### 2.9 Statistical analysis

All data were presented as mean ± SEM unless otherwise indicated. Statistical analyses were conducted using the GraphPad Prism software version 9.2.0. Pairwise comparisons between two groups were assessed for normality using the Shapiro–Wilk test. Datasets that passed were analyzed using an unpaired Student’s t-test with Welch’s correction, while datasets that did not pass were analyzed using the Mann–Whitney rank-sum test. Two-way ANOVA was used to analyze the plaque area versus distance graphs. Differences were considered statistically significant at p ≤ 0.05.

## 3 Results

### 3.1 Effects of *Il-15* knockout on atherosclerosis in *ApoE^−/−^* mice

**Figure 1:**
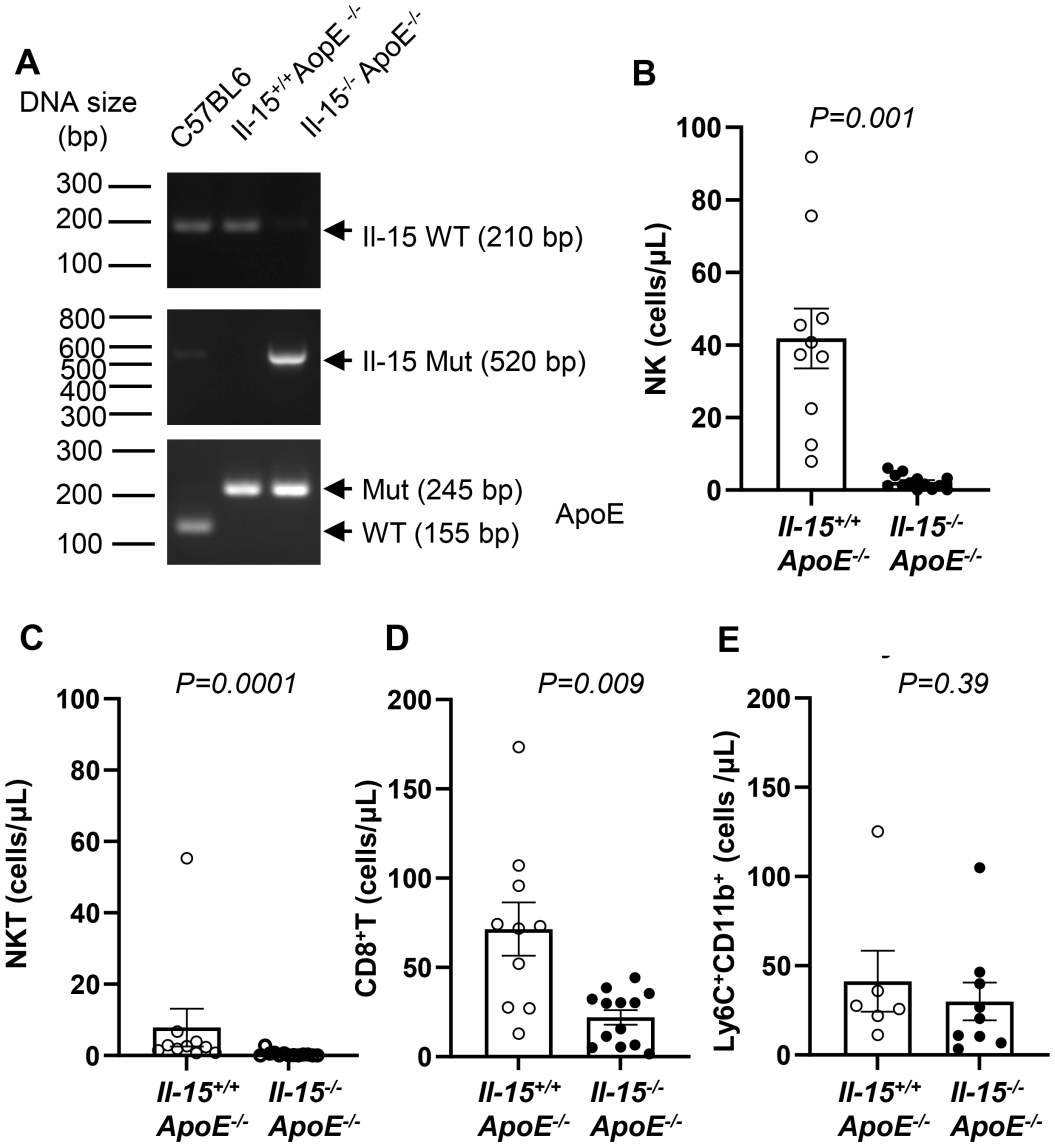
Circulating blood cell populations and inflammatory markers in *ApoE^−/−^* mice with reduced *Il-15* gene expression. **(A)** Representative PCR genotyping analysis of Il-15 and ApoE wild type (WT; +) and knockout mutant (Mut; −) alleles in samples from C57BL6 (Il-15^+/+^ApoE^+/+^), Il-15^+/+^ApoE^−/−^, and Il-15^−/−^ApoE^−/−^ mice. Numbers on the left indicate the positions of migration of DNA size standards on the same gels. Il-15 WT (210 bp), Mut (520 bp), ApoE WT (155 bp), and Mut (245 bp) are shown on the left. **(B)** Natural killer (NK), **(C)** NK T, **(D)** CD8^+^ T, and **(E)** Ly6C^+^CD11b^+^ cell concentrations in blood were determined via flow cytometry using FITC-labeled CD3 and PE-labeled NK1.1 antibodies **(B, C)**, FITC-labeled CD3 and APC-H7-labeled CD8 antibodies **(D)**, and APC-labeled Ly6C and FITC-labeled CD11b antibodies **(E)**. N = 6–13 mice per group, where each point represents an individual mouse. Bars are means ± SEM. p-Values are indicated on the graphs. Data in **(A, C)** passed the Shapiro–Wilk test for normality and were analyzed using an unpaired Student’s t-test with Welch’s correction.

**Figure 2:**
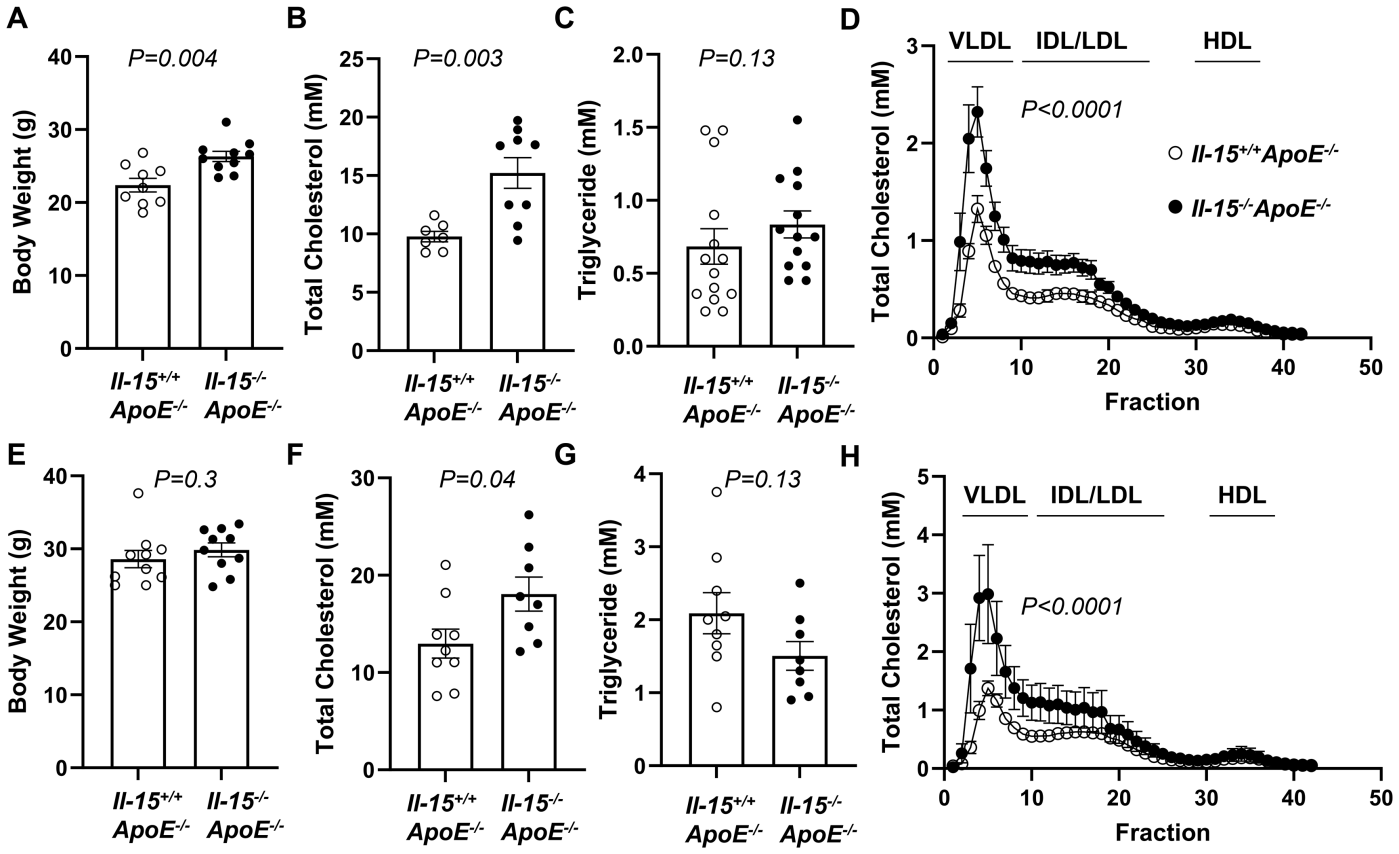
Body weights and plasma lipids in *ApoE^−/−^* mice with reduced *Il-15* gene expression. Female **(A–D)** and male **(E–H)** mice fed a normal diet were analyzed at 25 weeks of age. N = 8–10 mice per group, where each data point represents a different mouse. Body weights **(A, E)**, plasma total cholesterol **(B, F)**, and triglyceride levels **(C, G)** and plasma lipoprotein total cholesterol profiles **(D, H)** are shown. Data in **(A, B, F, G)** passed the Shapiro–Wilk test for normality and were analyzed using an unpaired Student’s t-test with Welch’s correction. Data in **(C, H)** did not pass the Shapiro–Wilk test for normality and were analyzed using the Mann–Whitney rank-sum test. Data in **(D, H)** were analyzed using two-way ANOVA.

To investigate the influence of IL-15 on atherosclerosis, we examined the consequences of inactivating the *Il-15* gene expression by crossing *Il-15^−/−^* and *ApoE^−/−^* mice to generate *Il-15^−/−^ApoE^−/−^* mice (**Figure 1A**). As expected from previous analyses of *Il-15*-deficient mice (Kennedy et al., 2000; Ashkar et al., 2003; Ashkar et al., 2009; Barra et al., 2010; White et al., 2017), elimination of *Il-15* expression was associated with reductions in the levels of circulating NK (2.3 ± 0.5 versus 41.8 ± 8.2 cells/μL, p = 0.001), NK T (0.43 ± 0.2 versus 7.9 ± 5.3 cells/μL, p = 0.0001), and CD8^+^ T cells (22.1 ± 4.1 versus 71.5 ± 14.9 cells/μL, p = 0.009) for *Il-15^−/−^ApoE^−/−^* versus *Il-15^+/+^ApoE^−/−^* mice (**Figures 1B–D**). There were no differences in the abundance of CD11b^+^ Ly6C^high^ monocytic cells in blood (**Figure 1E**). Knockout of *Il-15* was associated with a slight increase in body weights in female (**Figure 2A**; 26 ± 0.7 versus 22 ± 0.9 g, p = 0.004) mice, consistent with our previous findings (Barra et al., 2010; Barra et al., 2014), but did not affect body weights of male *ApoE^−/−^* mice (**Figure 2E**). Plasma total cholesterol was elevated in both female (**Figure 2B**; 15 ± 1.3 versus 9.8 ± 0.45 mM, p = 0.003) and male (**Figure 2F**; 12 ± 1.7 versus 7.6 ± 1.5 mM, p = 0.04) *Il-15^−/−^ApoE^−/−^* compared to *Il-15^+/+^ApoE^−/−^* mice; however, triglyceride levels were not statistically significantly different (**Figures 2C, G**). Lipoprotein size fractionation revealed that the elevated total cholesterol was primarily associated with very low density lipoprotein (VLDL)-sized lipoproteins (**Figures 2D, H**; p < 0.0001).

**Figure 3:**
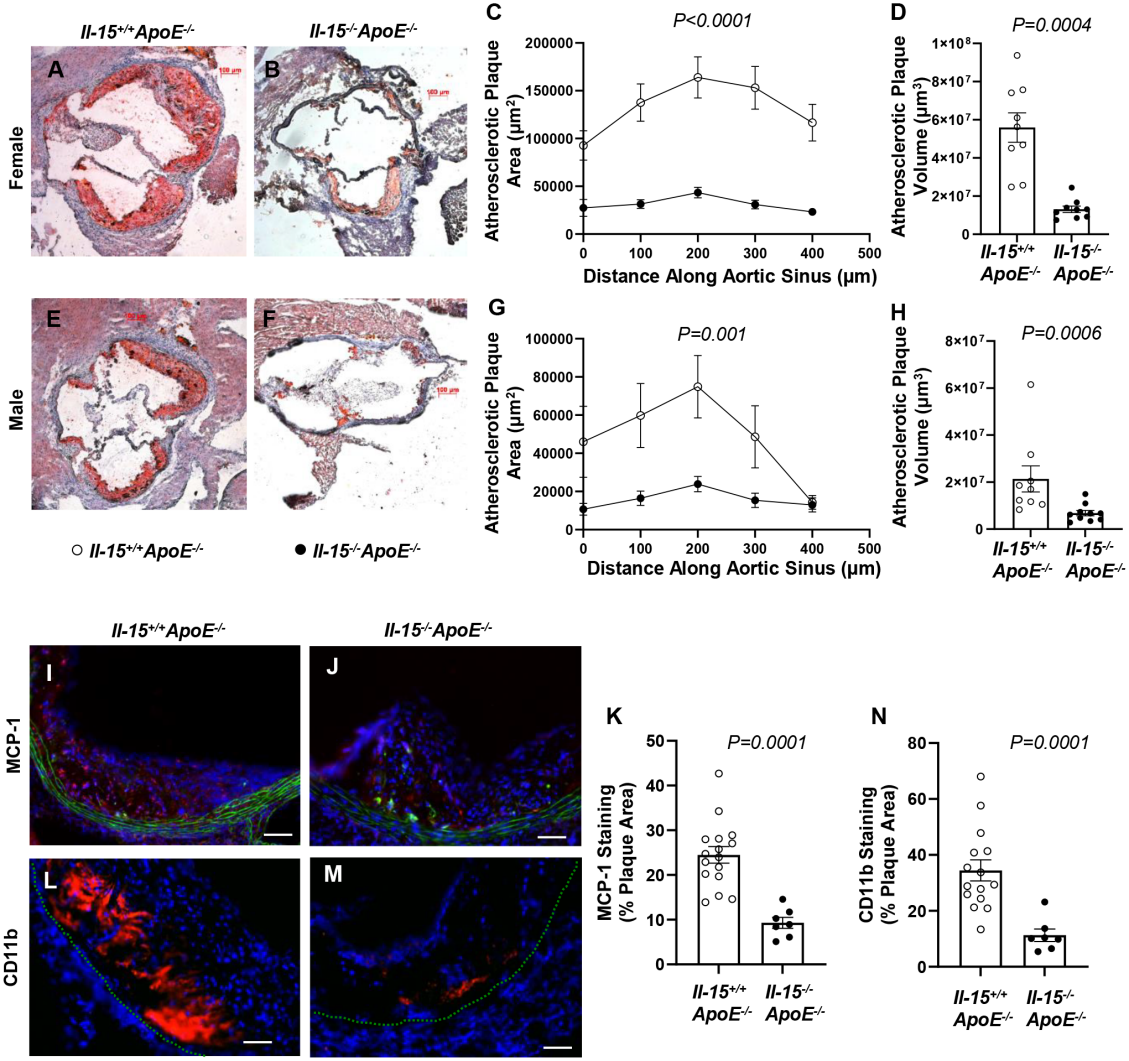
*Il-15* deficiency reduces atherosclerosis in normal diet-fed 25-week-old female and male *ApoE*^−/−^ mice. Representative images of atherosclerotic plaques [**(A, B, E, F)**; scale bars represent 100 µm], atherosclerotic plaque cross-sectional areas versus distance **(C, G)**, and atherosclerotic plaque volumes in the aortic sinuses **(D, H)** of female **(A–D)** and male **(E–H)** *Il-15^+/+^ApoE^−/−^* and *Il-15^−/−^ApoE^−/−^* mice. Immunofluorescence staining for MCP-1 [**(I, J)**; MCP-1 in red, DAPI staining for nuclei in blue, and green autofluorescence staining showing elastin fibers] and CD11b [**(L, M)**; CD11b in red and DAPI in blue]. Dotted lines show boundary of the artery wall and plaques from female *Il-15^+/+^ApoE^−/−^* and *Il-15^−/−^ApoE^−/−^* mice (scale bar represents 20 µm). Quantification of MCP-1 **(K)** and CD11b staining **(N)** expressed as % plaque area; each point represents a section from a different individual mouse. Data in **(C, G)** represent averages ± SEM of 9–10 mice per group and were analyzed using two-way ANOVA with Holm–Sidak post-test. Data in **(D, K, N)** passed the Shapiro–Wilk test for normality and were analyzed using an unpaired Student’s t-test with Welch’s correction. Data in **(H)** did not pass the Shapiro–Wilk test for normality and were analyzed using the Mann–Whitney rank-sum test. p-Values are indicated above each graph.

Atherosclerotic plaque sizes in 25-week-old female (**Figures 3A–D**) and male (**Figures 3E–H**) *Il-15^−/−^ApoE^−/−^* mice fed a normal, low-fat, low-cholesterol diet were significantly reduced compared to those in control *Il-15^+/+^ApoE^−/−^* mice (**Figures 3D, H**; females, 13.1 ± 1.7 × 10^6^ μm^3^ versus 55.9 ± 7.7 × 10^6^ μm^3^, p = 0.0004, respectively; males, 6.8 ± 1.2 × 10^6^ μm^3^ versus 21.3 ± 5.5 × 10^6^ μm^3^, p = 0.0006, respectively). Immunofluorescence staining of plaques in the aortic sinuses of female mice revealed that inactivation of Il-15 expression was associated with reduced protein levels of MCP-1 within atherosclerotic plaques (**Figures 3I–K**; 9.3% ± 3.2% versus 24.5% ± 7.4% of the plaque cross-sectional area for *Il-15^−/−^ApoE^−/−^* compared to *Il-15^+/+^ApoE^−/−^* mice). Consistent with the reduced levels of MCP-1, plaques from female *Il-15^−/−^ApoE^−/−^* mice contained fewer monocytic cells, based on reduced CD11b staining (**Figures 3L–N**; 11.3% ± 5.9% versus 34.5% ± 14.7% of the plaque cross-sectional area for *Il-15^−/−^ApoE^−/−^* compared to *Il-15^+/+^ApoE^−/−^* mice).

**Figure 4:**
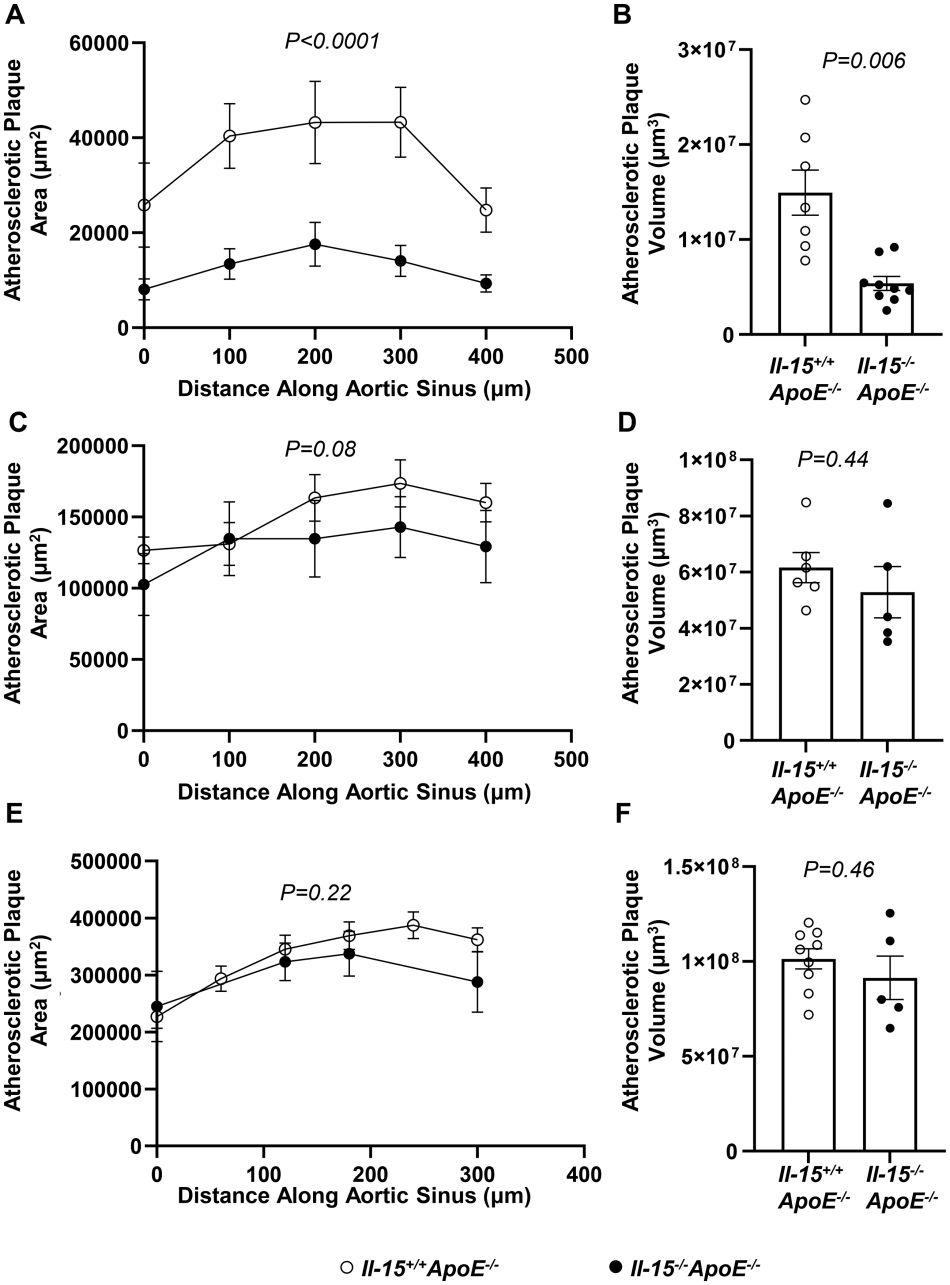
Impacts of age and diet on protection of female *ApoE*^−/−^ mice from atherosclerosis by *Il-15* deficiency. Atherosclerotic plaque cross-sectional areas versus distance **(A, C, E)** and atherosclerotic plaque volumes in the aortic sinuses **(B, D, F)** of female *Il-15^+/+^ApoE^−/−^* and *Il-15^−/−^ApoE^−/−^* mice fed a normal diet and analyzed at 15 **(A, B)** or 38 weeks of age **(C, D)** or mice fed a high-fat diet for 15 weeks and analyzed at 25 weeks of age **(E, F)**. Data in **(A, C, E)** represent averages ± SEM of 5–9 mice per group and were analyzed using two-way ANOVA with Holm–Sidak post-test. Data in **(B, D, F)** passed the Shapiro–Wilk test for normality and were analyzed using an unpaired Student’s t-test with Welch’s correction. p-Values are indicated above each graph.

To more comprehensively examine the impacts of *Il-15* knockout on atherosclerosis, we examined additional time points (15 and 38 weeks of age) of spontaneous atherosclerosis development in female *Il-15^−/−^ApoE^−/−^* and *Il-15^+/+^ApoE^−/−^* mice (**Figure 4**). As expected, atherosclerotic plaques were smaller in 15-week-old (**Figure 4A**) than 25-week-old (**Figure 3A**) female *Il-15^+/+^ApoE^−/−^* mice. Consistent with results in 25-week-old mice, plaques were considerably smaller in 15-week-old female *Il-15^−/−^ApoE^−/−^* than corresponding *Il-15^+/+^ApoE^−/−^* mice (**Figures 4A, B**; 5.0 ± 0.6 × 10^6^ μm^3^ versus 14.1 ± 2.6 × 10^6^ μm^3^, p = 0.02). In contrast, atherosclerotic plaque sizes were not different in 38-week-old female *Il-15^−/−^ApoE^−/−^* versus *Il-15^+/+^ApoE^−/−^* mice (**Figures 4C, D**; 53 ± 9 × 10^6^ μm^3^ versus 61 ± 5 × 10^6^ μm^3^; p = 0.44), suggesting that knockout of *Il-15* delayed but did not prevent spontaneous atherosclerosis in *ApoE^−/−^* mice. To determine if *Il-15* knockout impacted high-fat, high-cholesterol diet-accelerated atherosclerosis development, we fed female *Il-15^−/−^ApoE^−/−^* and *Il-15^+/+^ApoE^−/−^* mice a high-fat, high-cholesterol diet containing sodium cholate starting at 10 weeks of age and continuing for 15 weeks. Under these conditions, atherosclerotic plaque sizes were not statistically significantly different between *Il-15^−/−^ApoE^−/−^* and *Il-15^+/+^ApoE^−/−^* mice (**Figures 4E, F**; 91 ± 11 × 10^6^ μm^3^ versus 101 ± 5 × 10^6^ μm^3^; p = 0.46). Together, these data suggest that protection afforded by *Il-15* gene inactivation could be overcome by extending the time of atherosclerosis development or by atherogenic diet feeding.

### 3.2 Antibody-mediated neutralization of IL-15 reduces atherosclerosis in *ApoE^−/−^* mice

**Figure 5:**
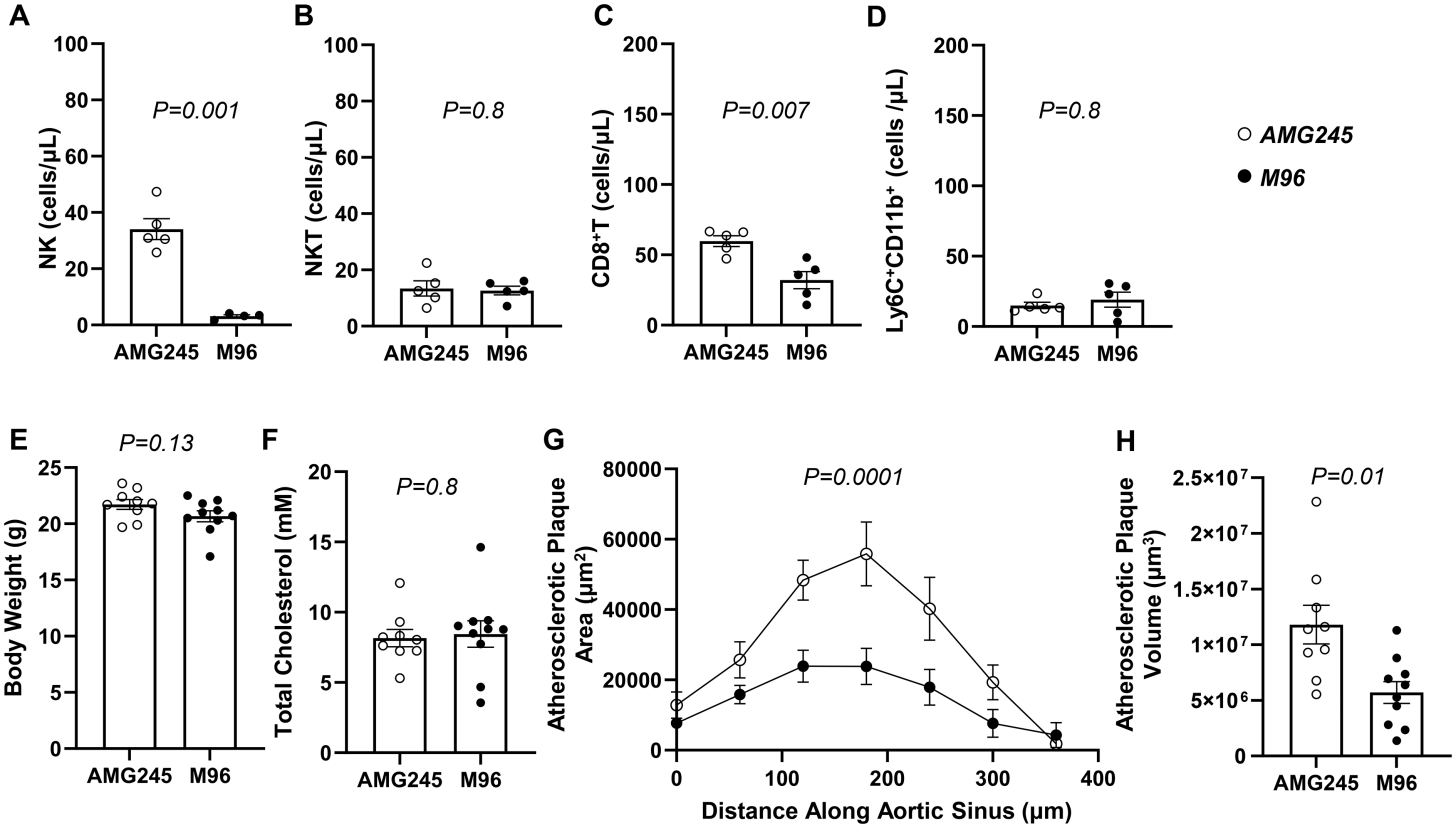
Antibody-mediated neutralization of IL-15 reduces spontaneous atherosclerosis in female *ApoE^−/−^* mice. Female 9-week-old *ApoE^−/−^* mice were treated weekly with 25 μg of either an anti-IL-15 mAb (M96) or an isotype control mAb (AMG245) until they were 15 weeks of age. Mice were fed a normal diet throughout. Concentrations of **(A)** natural killer (NK), **(B)** NK T, **(C)** CD8^+^ T, and **(D)** Ly6C^+^CD11b^+^ cells were determined via flow cytometry. **(E)** Body weights of mice at harvest. **(F)** Plasma total cholesterol levels. **(G)** Atherosclerotic plaque cross-sectional areas were determined at different distances along a 350-μm stretch of the aortic sinus from the aortic root. **(H)** Atherosclerotic plaque volumes were determined as the areas under the curves of plaque cross-sectional area versus distance for each mouse. Each data point in **(A–F, H)** represents an individual mouse, and bars with error bars represent means ± SEM. Each point in **(G)** represents the mean, and error bars represent SEM for groups of n = 8 mice treated with control IgG AMG245 and n = 10 mice treated with anti-IL-15 IgG M96. Data in **(A–F, H)** were analyzed for normality using the Shapiro–Wilk test. Those that passed **(A–C, E, F, H)** were analyzed using Student’s t-test with Welch’s correction. Those that did not pass the normality test **(D)** were analyzed using the Mann–Whitney rank-sum test. Data in **(G)** were analyzed using two-way ANOVA with Holm–Sidak post-test. p-Values are indicated on each graph.

We next treated female *ApoE^−/−^* mice, beginning at 9 weeks of age, with a mouse mAb (M96) that was previously reported to block the binding of IL-15 to the IL-2Rβγ_c_ complex without affecting IL-15 binding to IL-15Rα (Lebrec et al., 2013). As a control, a separate group of age-matched female *ApoE^−/−^* mice was treated with the same dose of a control mouse mAb, AMG245. Mice were treated weekly for 6 weeks with 25 µg of the M96 or control mAb and maintained on the normal diet. This dose was sufficient to decrease NK cells by 90% (**Figure 5A**; 3.1 ± 0.5 versus 34.1 ± 3.7 cells/μL; p = 0.001) and CD8^+^ T cells by 45% (**Figure 5C**; 32 ± 6 versus 59.8 ± 3.8 cells/μL, p = 0.007), while NK T cells and CD11b^+^Ly6C^high^ cells were unchanged (**Figures 5B, D**). Body weights and plasma total cholesterol levels were also unchanged in M96 anti-IL-15 mAb- versus control mAb-treated mice (**Figures 5E, F**). Treatment with the anti-IL-15 mAb did, however, substantially reduce atherosclerotic plaque sizes in the aortic sinus (**Figures 5G, H**; 5.3 ± 0.9 × 10^6^ μm^3^ versus 11.1 ± 1.6 × 10^6^ μm^3^, p = 0.01), consistent with the impacts of *Il-15* knockout on atherosclerosis in 15-week-old mice.

### 3.3 Targeting IL-15 reduces the accumulation of CD8^+^ cells in the artery wall

**Figure 6:**
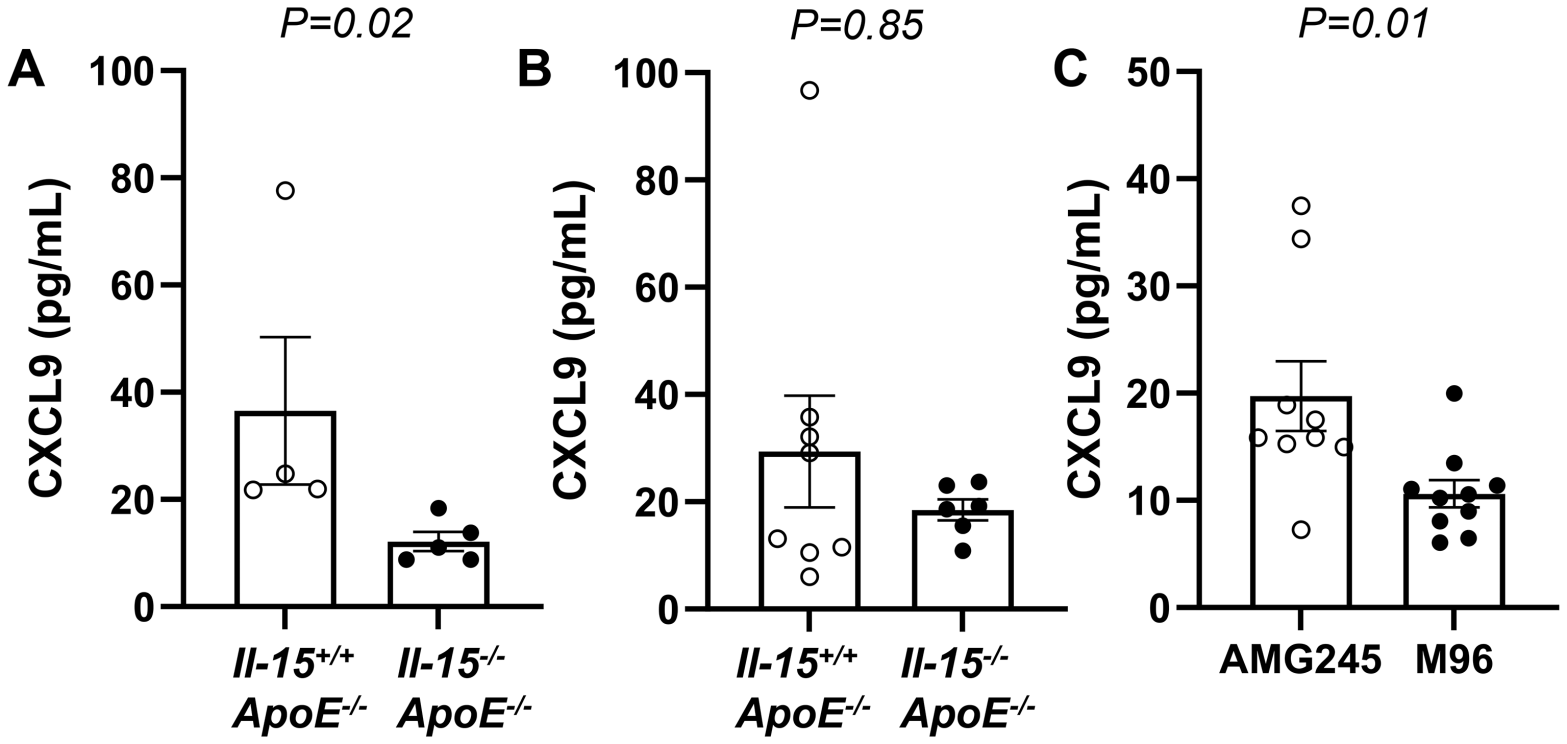
Impact of *Il-15* gene inactivation or antibody neutralization on plasma CXCL9 levels in *ApoE^−/−^* mice. Plasma was collected from either **(A)** male or **(B)** female *Il-15^−/−^ApoE^−/−^* and control *Il-15^+/+^ApoE^−/−^* mice at 25 weeks of age or **(C)** female *ApoE^−/−^* mice at 15 weeks of age that had been treated with the anti-IL-15 neutralizing (M96) or isotype control (AMG245) mAb for 6 weeks. CXCL9 levels were analyzed as part of the Mouse Cytokine Array 32-Plex by Eve Technologies (see **Supplementary Table**). Individual data points represent data from individual mice. Bars and error bars represent means ± SEM. Datasets did not pass the Shapiro–Wilk test for normality and were analyzed using the Mann–Whitney rank-sum test. p-Values are indicated above each graph.

**Figure 7:**
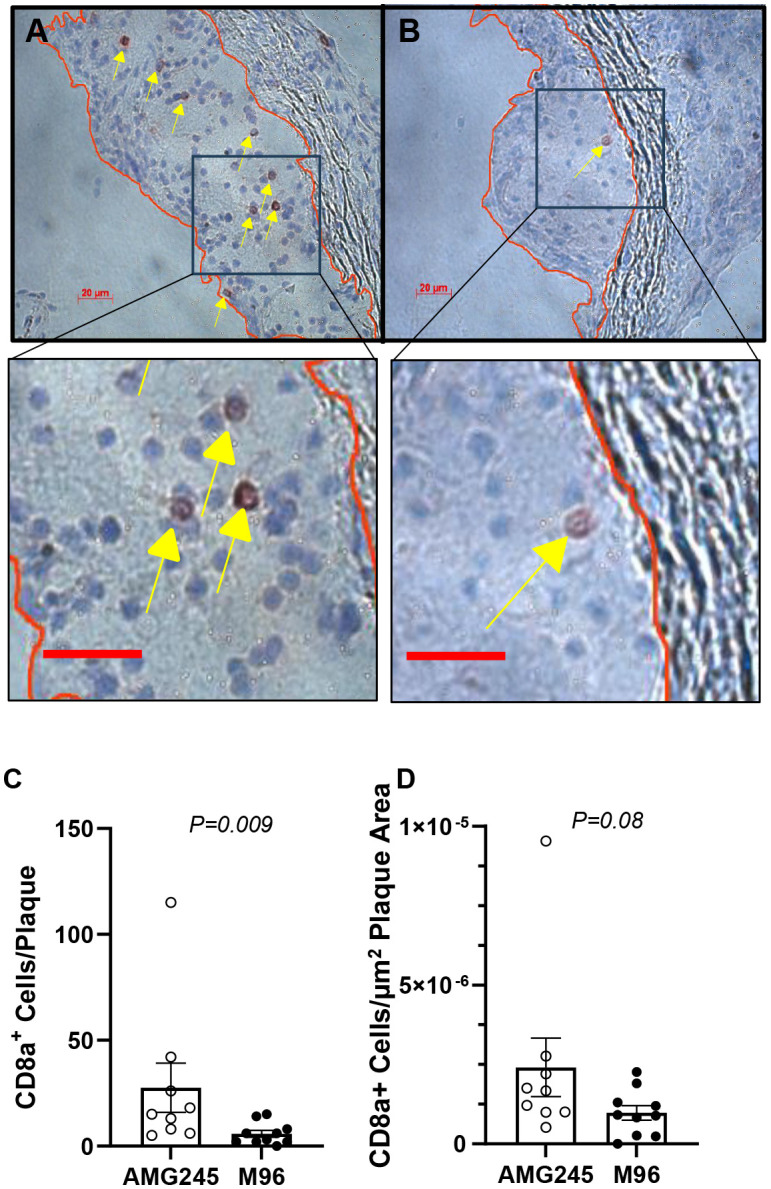
Impact of anti-IL-15 antibody-mediated neutralization on CD8a^+^ cells in the atherosclerotic artery walls of *ApoE^−/−^* mice. Representative images of immunostaining for CD8a in atherosclerotic plaques in the aortic sinus walls of **(A)** control mAb AMG245-treated and **(B)** anti-IL-15 M96 mAb-treated female *ApoE^−/−^* mice (mice were treated weekly for 6 weeks and harvested at 15 weeks of age as described in the legend to **Figure 5**). Higher-magnification images of the boxed areas are shown below. Red lines delineate the borders of atherosclerotic plaques, and yellow arrows indicate CD8a^+^ cells. Scale bars represent 20 µm. **(C)** Numbers of CD8a^+^ cells in atherosclerotic plaques were counted in one section of the aortic sinus per mouse. **(D)** Numbers of CD8a^+^ cells in atherosclerotic plaques normalized to the cross-sectional area of the corresponding atherosclerotic plaque in each sample. Each data point represents an individual mouse. Bars and error bars represent means ± SEM. Data did not pass the Shapiro–Wilk test for normality and were analyzed using the Mann–Whitney rank-sum test [p-value indicated on the graphs in **(C, D)**].

To evaluate the impact of targeting IL-15 on systemic inflammation, plasma from normal diet-fed male and female *Il-15^−/−^ApoE^−/−^* and *Il-15^+/+^ApoE^−/−^* mice and from anti-IL-15 (M96) mAb and control (AMG245) mAb-treated female *ApoE^−/−^* mice was subjected to multiplex analysis of the levels of cytokines and chemokines (**Supplementary Table 1**). Of the chemokines and cytokines tested, only CXCL9 showed consistent trends toward lower levels in the plasma of mice in which IL-15 was targeted. Average CXCL9 levels were reduced by approximately 67% in male *Il-15^−/−^ApoE^−/−^* mice when compared to *Il-15^+/+^ApoE^−/−^* mice (**Figure 6A**: 12.2 ± 1.8 pg/mL versus 36.5 ± 13.7 pg/mL, p = 0.02). Average CXCL9 levels showed a similar trend toward reduction, which did not reach statistical significance in female *Il-15^−/−^ApoE^−/−^* compared to *Il-15^+/+^ApoE^−/−^* mice (**Figure 6B**; 18.5 ± 2.0 pg/mL versus 29.4 ± 9.8 pg/mL, p = 0.9). Similarly, average CXCL9 levels were reduced by approximately 46% in anti-IL-15 (M96) mAb-treated compared to control (AMG245) mAb-treated female *ApoE^−/−^* mice (**Figure 6C**; 10.6 ± 1.3 pg/mL versus 19.7 ± 3.3 pg/mL, p = 0.01). When the data were expressed as % of control levels and pooled, the plasma CXCL9 levels were reduced by 48% in mice in which IL-15 was either inactivated or blocked (**Supplementary Table 1**; p = 0.004); however, this did not meet the threshold of statistical significance (0.0001) with the application of the Bonferroni correction for multiple (31) analyses. Consistent with a reported role for CXCL9 in the tissue recruitment of cytotoxic T cells (Ochiai et al., 2015; Ding et al., 2016), staining for CD8a, a marker of cytotoxic T cells, revealed reduced accumulation of CD8^+^ cells in the atherosclerotic artery wall of the aortic sinuses of female *ApoE^−/−^* mice treated with the anti-IL-15 (M96) mAb compared to the control (AMG245) mAb (**Figure 7C**; 5.8 ± 1.6 versus 27.5 ± 11.6 CD8^+^ cells per aortic sinus cross-section, p = 0.01). When the numbers of CD8^+^ cells were normalized to the total atherosclerotic plaque cross-sectional area, the difference was diminished and did not reach statistical significance, although a similar trend was observed (**Figure 7D**; 0.98 ± 0.22 versus 2.4 ± 0.9 × 10^−6^ cells/µm^2^).

## 4 Discussion

IL-15, a pro-inflammatory cytokine crucial for the homeostasis and function of CD8^+^ T cells and NK cells, has been detected in atherosclerotic plaques in both experimental mice and humans (Wuttge et al., 2001; van der Meer et al., 2011; Panigrahi et al., 2020). In human atherosclerotic plaques, the presence of IL-15 has been associated with increased CX3CL-1 levels and CD8^+^ cells in atherosclerotic plaques (Panigrahi et al., 2020). In this study, we investigated the effects of *Il-15* gene inactivation and antibody-mediated neutralization of IL-15 on atherosclerosis in *ApoE^−/−^* mice. Our findings demonstrate that *Il-15* deletion reduced spontaneous atherosclerosis by 70%–75% in male and female 15- and 25-week-old *ApoE^−/−^* mice. However, this protective effect was not observed in older, 38-week-old female *ApoE^−/−^* mice fed the normal diet or in 25-week-old female *ApoE^−/−^* mice that had been fed a high-fat, high-cholesterol diet for 15 weeks to accelerate atherosclerosis development. Therefore, *Il-15* deletion appeared to delay but did not fully inhibit spontaneous atherosclerosis development and did not impact diet-accelerated atherosclerosis development, at least at the time point measured in female mice. A similar pattern has previously been reported with *Il-10* deletion in *ApoE^−/−^* mice; while *Il-10* deficiency increased atherosclerosis at 16 weeks, no differences in plaque size were detected between *Il-10^−/−^ApoE^−/−^* and control *ApoE^−/−^* mice by 48 weeks of age (Caligiuri et al., 2003).

Previous studies have shown that *il-15* overexpression reduces while *il-15* deletion increases adiposity and body weight in mice fed normal diets (Barra et al., 2010; Barra et al., 2012; Barra et al., 2014), although others have reported that Il-15 deficiency attenuates high fat diet-induced obesity (Lacraz et al., 2016). In this study, we observed an approximately 10% increase in body weight following *Il-15* deletion in 25-week-old female *ApoE^−/−^* mice, similar to our previous analysis of the impact of Il-15 deficiency on body weight in otherwise wild-type mice on a C57BL/6 background (Barra et al., 2010). However, no significant differences in body weights were observed between male *Il-15^−/−^ApoE^−/−^* mice or in anti-IL-15 (M96) mAb-treated *ApoE^−/−^* mice. This lack of change in body weight may be due to the reported resistance of *ApoE^−/−^* mice to weight gain (Karagiannides et al., 2008). We also observed that *Il-15* deficiency increased plasma cholesterol levels in normal diet-fed *ApoE^−/−^* mice and that this was due to increased cholesterol associated with VLDL-sized lipoproteins. However, IL-15 inactivation with the M96 mAb did not result in a similar increase in plasma cholesterol, perhaps suggesting that the impact of IL-15 on plasma cholesterol may be independent of its interaction with IL-2Rβγ_c_. Alternatively, it is possible that the reduced timeframe of IL-15 inactivation in the M96 antibody-treated mice (7 weeks) compared to the mice in which Il-15 expression was inactivated genetically may explain the lack of an effect on weight gain and plasma cholesterol levels. Finally, it remains possible that the increased weight gain in females and the increased plasma cholesterol levels in both male and female *Il-15^−/−^apoE^−/−^* compared to *Il-15^+/+^apoE^−/−^* mice fed a normal diet may be the result of developmental compensation. Further studies will be required to uncover the mechanism by which *Il-15* deficiency increased VLDL-cholesterol levels, which remains to be determined. However, despite the increased VLDL-cholesterol levels, *Il-15* deficiency still reduced atherosclerotic plaque sizes in 25-week-old mice fed a normal diet, suggesting that IL-15 deficiency reduces early stages of atherosclerotic plaque size growth via pathways that can compensate, at least in part, for the increased plasma VLDL-cholesterol. These pathways appear to involve reduced MCP-1 protein levels and reduced CD11b^+^ monocytic cell accumulation in atherosclerotic plaques based on immunofluorescence analysis of plaques from 25-week-old female mice fed a normal diet. The extent to which these changes in plasma VLDL-cholesterol and plaque composition are maintained over time and contribute to the transient nature of the protection against atherosclerosis (15- and 25- but not 38-week-old mice fed the normal diet) or the lack of protection observed upon atherogenic diet feeding remains to be investigated.

Both *Il-15* deficiency and M96 mAb-mediated IL-15 neutralization resulted in a reduction of circulating CD8^+^ T cells and NK cells, both of which have been shown to promote atherosclerosis in mice in part through the secretion of perforin and granzyme B. Deletion of *Il-15* in males and M96 mAb-mediated neutralization of IL-15 in females were associated with trends toward reduced plasma levels of CXCL9, a chemokine associated with the recruitment of CD8^+^ T cells and NK cells into tissues (Ochiai et al., 2015; Ding et al., 2016; Fukuda et al., 2020). Since CD8^+^ T cells are among the most abundant lymphocytes in murine atherosclerotic plaques (Winkels et al., 2018; Zernecke et al., 2020; Barcia Durán et al., 2024), we stained atherosclerotic plaques for CD8^+^ cells and found that IL-15 neutralization significantly reduced the abundance of CD8^+^ cells in plaques of female *ApoE^−/−^* mice. These findings suggest that the protective effects against atherosclerosis, conferred by IL-15 inactivation, may be due, at least in part, to the reduced recruitment of CD8^+^ T cells to atherosclerotic plaques and are consistent with observations in human atherosclerotic plaques that IL-15 is associated with the recruitment of CD8^+^ cells into plaques (Panigrahi et al., 2020).

## 5 Limitations

Despite clearly demonstrating that targeting IL-15 reduces early stages of spontaneous atherosclerosis in ApoE-deficient mice, this study has important limitations. First, although the impact of IL-15 deficiency on the temporal progression of atherosclerotic plaque development was assessed, its impact on plasma cholesterol levels was evaluated at only a single time point (25 weeks old) in mice fed the normal chow diet. Therefore, it is not clear if the increased plasma total cholesterol levels in *Il-15^−/−^ApoE^−/−^* relative to *Il-15^+/+^ApoE^−/−^* mice persist at all ages or under high-fat diet feeding. If it does, then it is possible that the increased hypercholesterolemia accompanying IL-15 deficiency may eventually overcome the benefits of disrupting IL-15-mediated inflammatory pathways in older mice fed the normal chow diet or in mice fed the high-fat, high-cholesterol atherogenic diet. In this case, it may be that the impact on atherosclerosis reflects a balance between the protection afforded by reducing IL-15-mediated inflammation and the pro-atherogenic effects of increased hypercholesterolemia. It is possible that this balance favors reduced plaque formation at early stages of plaque growth in younger mice (15 and 25 weeks old fed the normal diet) but that this favorable balance is lost in older mice (38 weeks old) or in mice fed the atherogenic high-fat, high-cholesterol diet. Further detailed analysis of the dynamics of plasma cholesterol levels over the course of atherosclerotic plaque development, as well as the impacts of cholesterol-lowering in *Il-15^−/−^ApoE^−/−^* relative to *Il-15^+/+^ApoE^−/−^* mice, may help to resolve this.

Second, although we have analyzed the impacts of IL-15 inactivation on atherosclerotic plaque sizes and the abundance of CD8^+^ cells in the wall of the aortic sinus, the impacts on other aspects of atherosclerotic plaque composition have not been explored. Furthermore, we have analyzed the impacts of IL-15 knockout on atherosclerosis in 25-week-old ApoE^−/−^ mice fed the normal diet in both male and female mice but have explored the impacts at other ages (15 and 38 weeks old) or diets (high-fat, high-cholesterol diet containing cholate) and the impact of antibody-mediated inactivation in female mice only. We selected female mice, as they develop larger atherosclerotic plaques in the aortic sinus than male mice (**Figure 3**). Given that Il-15 deficiency had similar impacts on aortic sinus atherosclerosis in 25-week-old male and female ApoE^−/−^ mice, we think it is highly likely that Il-15 deficiency or antibody-mediated IL-15 blockade will similarly reduce spontaneous atherosclerosis in 15-week-old male mice. Whether, as in female mice, atherosclerosis in male mice is similarly unaffected by Il-15 knockout at older ages (e.g., 38 weeks of age) or when mice are fed an atherogenic high-fat, high-cholesterol diet remains to be determined.

Finally, we have examined the impacts of Il-15 gene inactivation or antibody blockade during initiation and active development stages of atherosclerosis. However, clinically, intervention is more frequently initiated under conditions of pre-existing atherosclerosis. Further studies, including detailed plaque compositional analysis beyond just atherosclerotic plaque size, would be required to determine if inactivation of IL-15 gene expression or M96 antibody-mediated blockade of IL-15’s interaction with the IL-2 Rβ/IL-2Rγ complex may impact pre-existing atherosclerosis.

## 6 Conclusion

Our findings suggest that IL-15 inactivation protects against early stages of spontaneous atherosclerosis in *ApoE^−/−^* mice by reducing circulating CD8^+^ T cells, decreasing CXCL9 levels, and limiting CD8^+^ T-cell recruitment to the artery wall, processes that are dependent on the interaction of IL-15 with the IL-2Rβγ_c_ complex. Their impacts on atherosclerotic plaque composition, beyond plaque size, on pre-existing atherosclerosis and the potential roles of other factors such as IL-15Rα warrant further study.

## Data Availability

The original contributions presented in the study are included in the article/**Supplementary Material**. Further inquiries can be directed to the corresponding author.
