# Supplementary material for: Inactivation of interleukin-15 reduces spontaneous atherosclerosis in apolipoprotein E-deficient mice
Source: Front Physiol. 2026 Aug 4;17:1859434. doi: 10.3389/fphys.2026.1859434 (PMC13481265; doi:10.3389/fphys.2026.1859434)
Supplement: Supplementary file 1 [file DataSheet1.pdf]

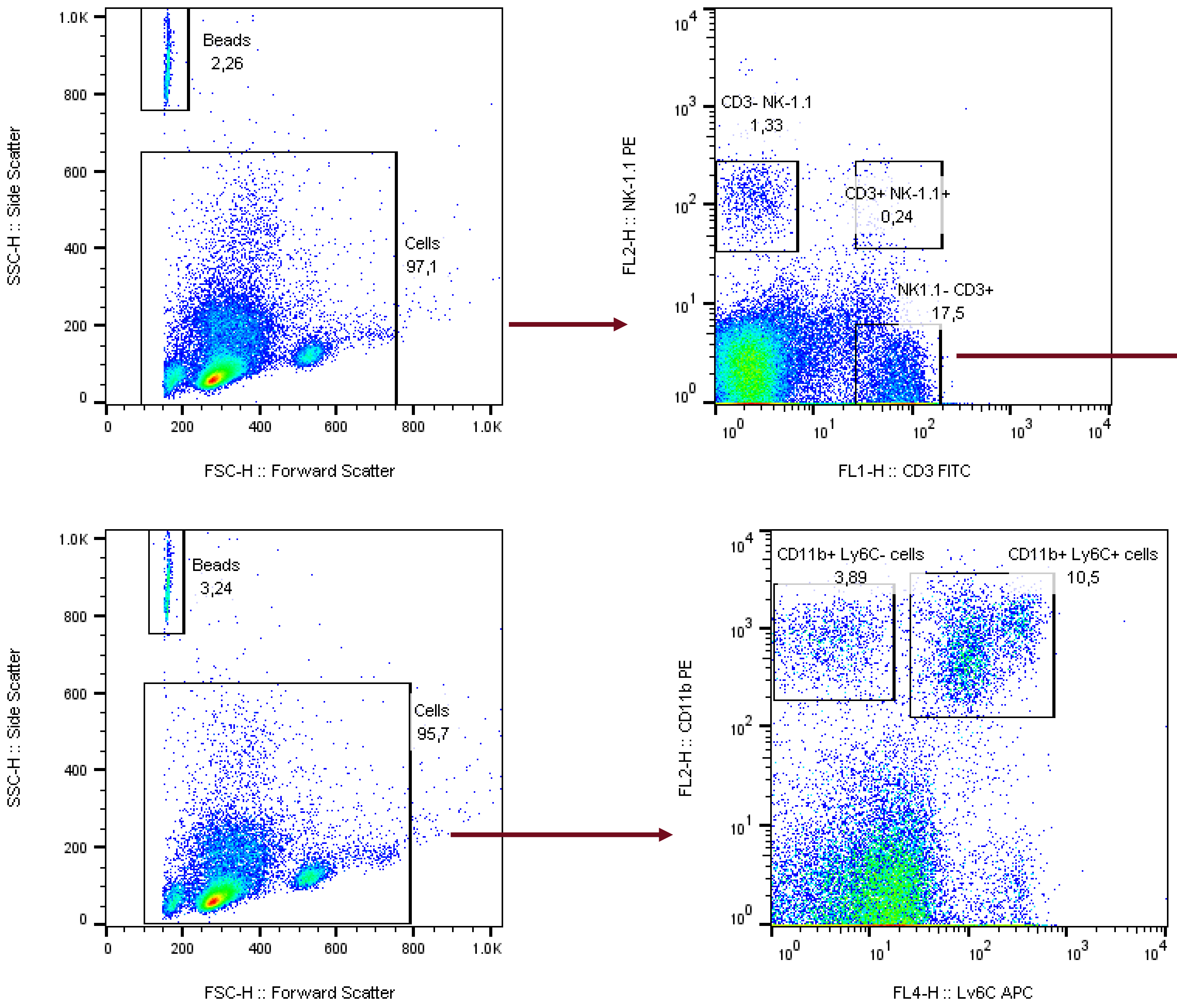

Supplementary Figure: Gating strategy for flow cytometry analysis of NK cells, NKT cells, CD8+T cells and CD11B+Ly6C+ monocytic cells.
