# Supplementary material for: Inactivation of interleukin-15 reduces spontaneous atherosclerosis in apolipoprotein E-deficient mice
Source: Front Physiol. 2026 Aug 4;17:1859434. doi: 10.3389/fphys.2026.1859434 (PMC13481265; doi:10.3389/fphys.2026.1859434)
Supplement: Supplementary file 2 [file Table1.docx]

|  | **Male** | | | **Female** | | | **Antibody treated female** | | | **Pooled (% control)** | | |
| --- | --- | --- | --- | --- | --- | --- | --- | --- | --- | --- | --- | --- |
| **Cytokine** | ***ApoE^-/-^*** | ***Il-15^-/-^ ApoE^-/-^*** | ***p-value*** | ***ApoE^-/-^*** | ***Il-15^-/-^ ApoE^-/-^*** | ***p- value*** | **AMG245** | **M96** | ***p- value*** | ***Control*** | ***IL-15 inactivated*** | ***p value*** |
| **CXCL9** | 36.5 ± 13.7 | 12.2 ± 1.8 | 0.02 | 29.4 ± 9.8 | 18.5 ± 2.0 | 0.9 | 19.7 ± 3.3 | 10.6 ± 1.3 | 0.01 | 100 ± 73 | 52 ± 20 | 0.004 |
| **CXCL10** | 139.5 ± 50.6 | 78.4 ± 23.3 | 0.6 | 52.7 ± 7.4 | 46.8 ± 5.8 | 0.5 | 57.6 ± 2.0 | 47.2 ± 6.0 | 0.1 | 100 ± 37 | 78 ± 33 | 0.1 |
| **Eotaxin** | 2782.6 ± 285.1 | 1976.4 ± 229.0 | 0.07 | 2223.6 ± 211.7 | 2234.8 ± 309.6 | 0.5 | 1683.5 ± 206.0 | 1055.5 ± 201.1 | 0.04 | 100 ± 26 | 75 ± 36 | 0.004 |
| **G-CSF** | 717.4 ± 84.6 | 257.3 ± 39.8 | 0.007 | 286.1 ± 29.5 | 277.5 ± 38.1 | 0.9 | 507.9 ± 48.8 | 456.5 ± 87.7 | 0.6 | 100 ±25 | 79 ± 48 | 0.03 |
| **GM-CSF** | - | - | - | 4.7 ± 7.8 | 15.6 ± 10.1 | 0.3 | 6.1 ± 2.9 | 4.4 ± 2.4 | 0.6 | ND | ND | ND |
| **IFN-γ** | - | - | - | 3.9 ± 2.9 | - | - | 0.3 ± 0.1 | 1.2 ± 0.7 | 0.8 | NF | ND | ND |
| **IL-1α** | 65.3 ± 23.7 | 34.0 ± 23.5 | 0.1 | 133.6 ± 80.1 | 77.7 ± 37.9 | 0.4 | 38.9 ± 7.8 | 32.0 ± 9.4 | 0.6 | 100 ± 115 | 68 ± 73 | 0.25 |
| **IL-1β** | 4.2 ± 2.2 | 46.7 ± 42.9 | 0.6 | 53.9 ± 30.0 | 32.3 ± 21.1 | 0.8 | 4.1 ± 1.3 | 5.8 ± 2.2 | 0.6 | 100 ± 120 | 350 ± 1000 | 0.4 |
| **IL-2** | 2.3 ± 0.1 | 2.9 ± 1.0 | 0.6 | 30.7 ± 18.6 | 2.7 ± 0.5 | 0.8 | 4.0 ± 0.8 | 4.4 ± 0.8 | 0.7 | 100 ± 110 | 87 ± 82 | 0.91 |
| **IL-3** | - | 1.3 ± 1.3 | - | 0.9 ± 0.4 | 0.6 ± 0.4 | 0.7 | 0.9 ± 0.2 | 0.6 ± 0.2 | 0.3 | 100 ± 110 | 96 ± 220 | 0.20 |
| **IL-4** | 0.4 ± 0.1 | 3.2 ± 2.5 | 0.2 | 5.8 ± 3.1 | 1.3 ± 0.4 | 0.6 | 1.1 ± 0.4 | 1.2 ± 0.5 | 0.6 | 100 ± 110 | 240 ± 700 | 0.9 |
| **IL-5** | 9.8 ± 1.4 | 20.0 ± 8.9 | 0.3 | 21.5 ± 8.7 | 23.9 ± 9.2 | 0.8 | 16.9 ± 3.5 | 16.2 ± 4.6 | 0.9 | 100 ± 82 | 126 ± 128 | 0.8 |
| **IL-6** | 10.5 ± 1.1 | 6.9 ± 2.6 | 0.3 | 10.3 ± 3.7 | 8.6 ± 1.5 | 0.8 | 6.1 ± 2.1 | 13.5 ± 4.1 | 0.07 | 100 ± 91 | 146 ± 166 | 0.35 |
| **IL-7** | 4.0 ± 1.2 | 15.4 ± 5.7 | 0.02 | 57.2 ± 34.3 | 11.6 ± 3.9 | 1.0 | 16.8 ± 11.4 | 5.7 ± 1.8 | 0.1 | 100 ± 170 | 114 ± 213 | 0.94 |
| **IL-9** | 58.0 ± 13.8 | 118.5 ± 49.9 | 0.4 | 120.5 ± 31.6 | 178.2 ± 21.2 | 0.2 | 100.9 ± 22.1 | 102.3 ± 18.7 | 0.9 | 100 ± 63 | 136 ± 98 | 0.24 |
| **IL-10** | 8.0 ± 3.0 | 36.4 ± 27.9 | 0.7 | 50.9 ± 26.1 | 33.9 ± 6.6 | 0.5 | 16.9 ± 5.7 | 21.0 ± 10.0 | 0.5 | 100 ± 115 | 190 ± 400 | 0.83 |
| **IL-12p40** | 21.7 ± 6.2 | 23.2 ± 11.3 | 0.9 | 4.3 ± 4.1 | 8.3 ± 5.6 | 0.6 | 8.5 ± 2.4 | 11.0 ± 3.2 | 0.8 | 100 ± 100 | 130 ± 190 | 0.92 |
| **IL-12p70** | 13.7 ± 12.7 | 233.1 ± 226.3 | 0.7 | 308.2 ± 165.6 | 105.6 ± 77.1 | 0.7 | 31.2 ± 16.1 | 53.4 ± 25.6 | 0.9 | 100 ± 150 | 497 ± 1800 | 0.92 |
| **IL-13** | 44.0 ± 7.2 | 49.3 ± 12.3 | 0.7 | 47.9 ± 15.8 | 38.5 ± 5.4 | 0.8 | 93.6 ± 13.1 | 39.0 ± 5.8 | 0.003 | 100 ± 65 | 69 ± 45 | 0.04 |
| **IL-17** | 1.7 ± 0.5 | 7.1 ± 4.6 | 0.8 | 12.0 ± 5.5 | 7.1 ± 4.8 | 0.6 | 2.4 ± 1.7 | 3.7 ± 2.2 | 0.6 | 100 ± 160 | 190 ± 360 | 0.81 |
| **KC** | 125.6 ± 54.1 | 41.8 ± 18.3 | 0.2 | 347.3 ± 168.5 | 133.9 ± 49.8 | 0.9 | 57.4 ± 19.5 | 39.5 ± 16.8 | 0.2 | 100 ± 112 | 51 ± 66 | 0.08 |
| **LIF** | 0.7 ± 0.2 | 1.3 ± 0.5 | 0.3 | 0.9 ± 0.2 | 1.1 ± 0.2 | 0.6 | 0.8 ± 0.1 | 1.6 ± 0.7 | 0.6 | 100 ± 49 | 168 ± 198 | 0.151 |
| **LIX53** | 1173.0 ± 209.9 | 1069.2 ± 347.8 | 0.8 | 887.2 ± 215.7 | 627.2 ± 48.5 | 0.3 | 1179.2 ± 336.3 | 2045.1 ±1058.3 | 0.5 | 100 ± 71 | 124 ± 199 | 0.803 |
| **MCP-1** | 4.7 ± 1.6 | 57.2 ± 46.5 | 0.1 | 73.5 ± 31.5 | 105.2 ± 34.4 | 0.5 | 17.9 ± 11.1 | 15.2 ± 7.9 | 1.0 | 100 ± 133 | 372 ± 1100 | 0.22 |
| **M-CSF** | 9.6 ± 2.0 | 8.1 ± 3.6 | 0.7 | 3.0 ± 5.2 | 12.1 ± 7.0 | 0.6 | 4.9 ± 1.3 | 7.1 ± 2.3 | 0.8 | 100 ± 124 | 207 ± 337 | 0.44 |
| **MIP-1α** | 25.5 ± 14.8 | 48.3 ± 26.2 | 0.5 | 169.7 ± 92.6 | 57.3 ± 12.2 | 0.5 | 44.8 ±13.1 | 60.3 ± 13.8 | 0.4 | 100 ± 119 | 119 ± 136 | 0.27 |
| **MIP-1β** | - | - | - | 27.8 ± 17.3 | - |  | 51.9 ± 14.0 | 25.4 ± 12.5 | 0.2 | 81 ± 128 | 31 ± 62 | 0.25 |
| **MIP-2** | 182.0 ± 123.3 | 107.4 ± 90.1 | 0.7 | 57.1 ± 66.3 | 196.3 ± 88.4 | 0.1 | 34.2 ± 12.6 | 105.2 ± 43.9 | 0.07 | 100 ± 109 | 259 ± 355 | 0.11 |
| **RANTES** | 17.0 ± 9.7 | 35.4 ± 14.9 | 0.3 | 80.6 ± 21.5 | 42.1 ± 11.3 | 0.2 | 41.8 ± 11.5 | 46.2 ± 13.8 | 1.0 | 100 ± 83 | 117 ± 127 | 0.99 |
| **TNF-α** | 2.9 ± 1.9 | 12.7 ± 9.1 | 0.6 | 17.3 ± 8.4 | 17.8 ± 7.3 | 0.5 | 3.1 ± 1.0 | 2.4 ± 0.8 | 0.7 | 100 ± 111 | 170 ± 357 | 0.69 |
| **VEGF** | 0.5 ± 0.1 | 0.4 ± 0.0 | 0.5 | 5.5 ± 3.2 | 0.5 ± 0.1 | 0.4 | 0.5 ± 0.1 | 0.6 ± 0.1 | 0.6 | 100 ± 105 | 74 ± 54 | 0.50 |

**Supplementary Table 1: Analysis of Plasma Cytokines in 25-Week-Old *Il-15^+/+^ApoE^-/-^* and *Il-15^-/-^ApoE^-/-^* Mice, and anti-IL-15 Antibody-Treated Female *ApoE^-/-^* Mice.** Plasma samples were analyzed using the Multiplex Immunoassay by Eve Technologies. Values are means ± standard deviations and are expressed in pg/ml except for the columns labeled “Pooled” which are a pool of the experiments (Males genetic inactivation , Females genetic inactivation and Antibod-mediated inactivation) expressed as % of control value (ApoE-/- male, female and female treated with control antibody). Concentrations falling outside the assay's detection range were excluded from the table, leaving corresponding entries blank. Statistical tests were conducted as outlined in the Methods section. Data following a normal distribution were analyzed using an unpaired t-test with Welch’s correction, while non-normally distributed data were analyzed using the Mann-Whitney test. P values are indicated. Application of the Bonferroni correction for multiple (31) analyses means that the threshold for statistical significance is 0.001. C-X-C motif ligand 9 (CXCL9) also known as monokine induced by gamma interferon(MIG), C-X-C motif cytokine ligand 10 (CXCL10) also known as Interferon gamma-induced protein 10 (IP-10), G-CSF: Granulocyte- colony stimulating factor, GM-CSF: Granulocyte- macrophage colony stimulating factor, IFN-γ: interferon- gamma, IL: Interleukin, KC: keratinocyte- derived cytokine, LIF: Leukemia inhibitory factor, LIX53: lipopolysaccharide- induced CXC chemokine 53, MCP-1: monocyte chemoattractant protein-1, M-CSF: macrophage colony stimulating factor, MIG74:, MIP: macrophage inhibitory protein, RANTES: regulated on activation, normal T cell expressed and secreted also known as C-C motif chemokine ligand 5 (CCL5), TNF-α: tumor necrosis factor alpha, VEGF: vascular endothelial growth factor.
